# Conceptual framework for a Danish human biomonitoring program

**DOI:** 10.1186/1476-069X-7-S1-S3

**Published:** 2008-06-05

**Authors:** Marianne Thomsen, Lisbeth E Knudsen, Katrin Vorkamp, Marie Frederiksen, Hanne Bach, Eva Cecilie Bonefeld-Jorgensen, Suresch Rastogi, Patrik Fauser, Teddy Krongaard, Peter Borgen Sorensen

**Affiliations:** 1 National Environmental Research Institute, University of Aarhus, Department of Policy Analysis, DK-4000 Roskilde, Denmark; 2 Institute of Public Health, University of Copenhagen, DK-1014 København K, Denmark; 3 National Environmental Research Institute, University of Aarhus, Department of Environmental Chemistry and Microbiology, DK-4000 Roskilde, Denmark; 4 Unit of Cellular & Molecular Toxicology, Institute of Public Health, University of Aarhus, DK-8000 Aarhus, Denmark; 5 National Environmental Research Institute, University of Aarhus, Department of Atmospheric Environment, DK-4000 Roskilde, Denmark; 6 National Environmental Research Institute, University of Aarhus, Department of Terrestrial Ecology, DK-8600 Silkeborg, Denmark

## Abstract

The aim of this paper is to present the conceptual framework for a Danish human biomonitoring (HBM) program. The EU and national science-policy interface, that is fundamental for a realization of the national and European environment and human health strategies, is discussed, including the need for a structured and integrated environmental and human health surveillance program at national level. In Denmark, the initiative to implement such activities has been taken. The proposed framework of the Danish monitoring program constitutes four scientific expert groups, i.e. i. Prioritization of the strategy for the monitoring program, ii. Collection of human samples, iii. Analysis and data management and iv. Dissemination of results produced within the program. This paper presents the overall framework for data requirements and information flow in the integrated environment and health surveillance program. The added value of an HBM program, and in this respect the objectives of national and European HBM programs supporting environmental health integrated policy-decisions and human health targeted policies, are discussed.

In Denmark environmental monitoring has been prioritized by extensive surveillance systems of pollution in oceans, lakes and soil as well as ground and drinking water. Human biomonitoring has only taken place in research programs and few incidences of e.g. lead contamination. However an arctic program for HBM has been in force for decades and from the preparations of the EU-pilot project on HBM increasing political interest in a Danish program has developed.

## Introduction

Sustainability and integrated protection of the environment and human health are closely linked [1]. Denmark has developed a national strategy for sustainable development for which the main goal is a constant decrease in pollutant levels in products, food, the working environment, traffic and the indoor environment [2-5]. The Danish vision of sustainable development is based on eight objectives and principles [2]:

*1. The welfare society must be developed and economic growth must be decoupled from environmental impacts*.

*2. There must be a safe and healthy environment for everyone, and we must maintain a high level of protection*.

*3. We must secure a high degree of bio-diversity and protect ecosystems*.

*4. Resources must be used more efficiently*.

*5. We must take action at an international level*.

*6. Environmental considerations must be taken into account in all sectors*.

*7. The market must support sustainable development*.

*8. Sustainable development is a shared responsibility and we must measure progress*.

Objective 2 is further described in the section on the cross-cutting issue 'Environment and health' which states that: *'Denmark should be a country where pollution from products, food, working environment, traffic and physical indoor conditions affecting the population's quality of life and health is constantly falling. Harm to animals and plants from pollution should also be limited. The protection level must take account of especially sensitive groups of people – such as children, pregnant women, people who suffer from allergies or from chronic illness – and of particularly vulnerable ecosystems' *[2].

In 2003, the European Commission launched the European Environment and Health Strategy [6,7]; a strategy to reduce diseases linked to environmental factors. The strategy, also known as SCALE, comprises the development of information systems as well as the compilation of adequate political measures. Its themes are: Scientific evidence, focus on Children, raising of Awareness, improving the situation by use of Legal instruments and allowing Evaluation of the progress made.

In the same year, Denmark published a background report [8] for a strategy and action plan to protect public health against environmental factors [9]. The strategy addresses chemicals with respect to their harmful effects, which is also addressed in the overall Danish chemicals strategy. The action plan includes a ten-point plan:

1. Negative impacts from chemicals are to be reduced, and the substitution of hazardous substances by less hazardous ones must be accelerated

2. The incidence of allergy and respiratory disorders is to be reduced

3. Measures directed at endocrine-disrupting substances are to be intensified

4. Noise nuisance is to be reduced

5. The negative impacts on health from air pollution and from the indoor climate are to be reduced

6. Food is to be safe and free from pollution

7. Groundwater and drinking water must be protected

8. Research into the significance of environmental factors on health is to be enhanced

9. Cooperation between the authorities must be strengthened

10. Increased attention must be accorded to environmental factors and health in international cooperation

Human biomonitoring is only addressed indirectly by the need for 'health monitoring'. Enhancement of the cooperation at administrative level is however highlighted as the 'National Board of Health has primary responsibility for general health monitoring, while the responsibilities of other ministries are more linked to preventive initiatives such as setting limit values and detailed requirements for the different sources of environmental factors'. Enhanced cooperation between ministries is to ensure coordinated and cohesive action against environmental factors that can affect health, and in particular within areas of common interest are needed for the realisation of the strategy and action plan [9].

The European Environment and Health Action Plan 2004–2010 stresses clearly the need for closer coordination between the health and environment research areas [10,11]. The action plan identifies 13 actions with a focus on: improving the information chain by developing integrated environment and health information (Action 1–4), filling the knowledge gap by strengthening research on environment and health and identifying emerging issues (Action 5–8) and reviewing and adjusting risk reducing policy and improve communication (Action 9–13) [12].

The ultimate goal of the European and the national strategies is to '*develop an environment and health cause-effect framework*' that will provide the necessary information for the development of policies dealing with sources and impact pathways of health stressors.

**Figure 1:**
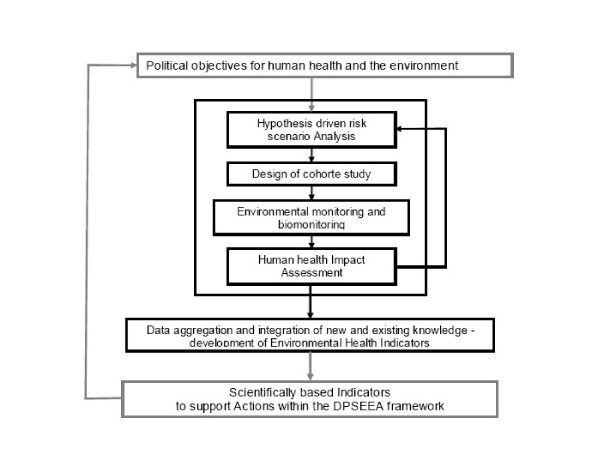
Framework for a national integrated environmental and human health surveillance program and relations to DPSEEA (Driving forces – Pressures – State – Exposure – Effects – Actions). The black box in the middle of the figure represents the national human biomonitoring program, which delivers outputs to support political targeted actions to protect human health and the environment. The science-policy interfaces are illustrated by the arrows connection the grey and black boxes.

Action 1 (Develop environmental health indicators) and Action 2 (Develop integrated monitoring of the environment, including food, to allow the determination of relevant human exposure) of the European Environment and Health Action Plan 2004–2010, concerns the health of the environment and integrated monitoring of environmental contamination leading to human exposure, i.e. external human exposure. Action 3, currently underway, focuses on internal human exposure or human biomonitoring. In the third action, the European Commission commits itself '*to develop in close cooperation with the Member States a coherent approach to Human Biomonitoring in Europe and to launch an EU Pilot Project to test out the feasibility of such a coordinated approach*'. For this reason, an Expert team to Support BIOmonitoring (ESBIO) together with the Implementation Group (IG) of the European HBM has been preparing implementation of an EU pilot project, which was launched in the spring 2007. The background and rationale for the EU Pilot Project and the Danish proposal of a conceptual framework for a national HBM program (cf. Figure 1) are presented in this paper. The proposed framework builds on the principles and experience gained from scientific work at national and EU level, e.g. NoMiracle, as well as environment and human health indicator reporting within the area of cumulative risk from exposure [7,13-19].

## Background

In the second recommendation from the Implementation Group (IG) and the final proposal for a European HBM project, two scenario types, the so-called 'basic' and 'extended' scenario, are described. The basic scenario includes mainly heavy metals (lead, mercury and cadmium) and the metabolite cotinine from nicotine in tobacco smoke, whereas the extended scenario includes contaminants, for example brominated flame retardants (BFRs), for which complex analytical methodologies are required.

Therefore, harmonization between countries may need to address both 1) quality assessment and assurance systems in relation to analytical chemistry and methodologies and 2) design and framework of the monitoring program for the quantification of state and development in exposure from stressors suspected to contribute to the priority diseases included in the national and European environment and health strategies. The two different levels of harmonization required in the European pilot project may be viewed in terms of:

• A basic scenario monitoring program, where the main purpose is to secure data quality and comparability in analytical methodologies across Europe, as well as to establish reference values or data ranges for the European population, enabling comparison according to exposure scenarios. Knowledge transfer between countries by means of proficiency testing systems for the participating laboratories in order to attain quality harmonization may be included here.

• An extended scenario, where the chemicals are selected based on a human health oriented framework approach and where the specific selection of chemicals may differ between countries, i.e. according to national priorities. Knowledge transfer between countries in order to develop a monitoring program designed to quantify trends and information needed to guide future environment and health priorities; i.e. an HBM framework including a science-policy interface, may be included here.

The basic scenario may not meet the requirements for national priorities for all member states due to the increased political concern directed at addressing high risk scenarios. Here, focus would be on high exposure potentials combined with high toxicity potentials in specific scenarios addressing specific priority diseases.

The extended scenario would call for a harmonisation process that has already been suggested in the WHO initiative for an integrated environment and health information system. From 2007, the Member States report to the WHO on the status and progress on the national activities regarding children's environment and health .

The process of obtaining consensus on the health issues and the selection criteria for chemicals to be monitored is central for the realization of the pilot project and in this respect some flexibility in interpretation of the word 'harmonization' may be useful. Harmonization may address the process in terms of the compounds to be monitored enabling quality assurance and proficiency testing arrangement between countries. Alternatively, harmonization may relate to development of a European framework for an approach to select biomarkers based on existing structures and knowledge on environmental quality and human health.

In order to meet the goals of the EU Environment and Health Strategy and the conditions formulated in SCALE, the European Environment Agency recommends that [6,7,20-23]:

• an information strategy for the program should be built on policy relevant indicators addressing the main policy questions [20,23]

• the focus of the monitoring and assessment efforts should be on relevant exposures to environmental factors [21,22]

• harmonized arrangements for managing monitoring information should be developed [7]

An example of a compound group which would belong to the extended scenario, and of broad international interest, is the BFRs which have been acknowledged as ubiquitous persistent pollutants concerning bioaccumulation potential and adverse health effects. Exponential increases have been found in human tissues since the 1970s, with indications of stagnation in European samples, possibly as a consequence of political regulation [24].

### Danish initiatives and projects

The Danish Environmental Research Institute publishes every fourth year a state of the environment report for Denmark, including a chapter on environment and health, presenting status and development in knowledge of the impact of environmental factor on human health [16]. Focussing on BFRs, existing knowledge of external and internal exposure was reviewed recently, with emphasis on identification of exposure routes [25]; the study concluded that unintentional ingestion of dust is at least of similar importance as food.

Analogous to e.g. the WHO's integrated information system ; a national pilot project on the integration of environmental data bases and population health registers has been performed at the national level, in Denmark. The project proposes that the geographically based health database of the National Board of Health, is utilised as the basis for linkage of health and environmental registers [26].

Several research projects are addressing chemicals and biomarkers focusing on the national priority diseases respiratory disorders and allergy included in the NEHAP [9]; e.g. COPSAC  and AIPOLIFE . Based on national collaborations, the Danish Board of Technology published a report, 'A better environment for children – a proposal for action' [in Danish], that recommended 14 actions to protect the health of children, including the need for '*coordinating of environmental and health guidance in relation to children's exposure to hazardous environmental factors' *and '*better systematic surveillance and risk assessment of the amount of environmentally related health hazardous substances in food, consumer products and drinking water' *[27]. Several other activities regarding children's health, e.g. research studies on biomarkers addressing childhood cancer and endocrine disruptors and on children's exposure to BFRs, have been completed or commenced.

As a follow-up on the Fourth Ministerial Conference on Environment and Health in Budapest, 23–25 June 2004 [23], the National Board of Health in Denmark published a collection of ideas regarding activities to improve children's environment and health in Denmark [28]. The ideas of this report have been presented to the Danish Interministerial Group for Environmental Factors and Health, which was established in relation to the National Environment and Health Action Plan (NEHAP). One of the ideas of the report are to establish a systematic surveillance and risk assessment of chemicals with negative effects on health in consumer products, food and tap water and to clarify the relation between environment and food contaminants and related health effects on male reproductive organs [28].

Integration of available information on environmental and human health trends may form the basis for a screening level indicator system that can be used to design a biomonitoring program. Thus, Environmental Health Indicators (EHI) identified scientifically are tools that summarize the relationship between the environment and health, and these can be used for regulatory actions to manage or prevent impacts on environment and health [13,14,17,23].

The proposal for a coordinated action involving existing infrastructures, experts within environment and health monitoring activities, and research for the support and implementation of a human biomonitoring program is an obvious continuation of the activities and cooperation that have already taken place among institutions in Denmark. Based on the experience with BFRs and quality assurance/quality control, Denmark has taken a leading role in the design of appropriate monitoring strategies and procedures of this compound group.

## Conceptual framework for a Danish HBM program

A HBM program should be a central part of any national integrated environmental and human health surveillance program, and there is a need to develop an interface between the science and the policy decision support systems – in SCALE denoted a 'response system'. To make this interface as transparent as possible, we suggest risk scenario descriptions and selections (prioritization) to form the basis for human health oriented biomonitoring surveys. The scenario selections will be based on hypothesis-driven risk scenario descriptions focusing on children as the most vulnerable sub-population. The scenario descriptions deliver input to the prioritization of the exposure modelling of high-risk chemicals with respect to the national and European priority diseases as well as the biomonitoring and monitoring activities. The modelling of outdoor exposure is already included in the national monitoring program of the environment [29], whereas the indoor exposure needs to be monitored in addition to internal exposure, i.e. biomarkers of exposure. This could be formulated as a minimum requirement for the output from the basic scenario, as defined in the third recommendation from the Implementation Group.

When focusing on biomarkers of exposure, as in the basic scenario of the proposed pilot project, it is crucial for the added value of the biomonitoring program that both exposure routes and related internal exposures are monitored. This will allow for the ratio of external to internal concentrations, i.e. C(external)/C(internal), by purpose of identifying science based 'safety-factors' with respect to long-tern low dose exposures and re-evaluation of environmental quality criteria protect human health from unacceptable aggregated exposures.

**Figure 2:**
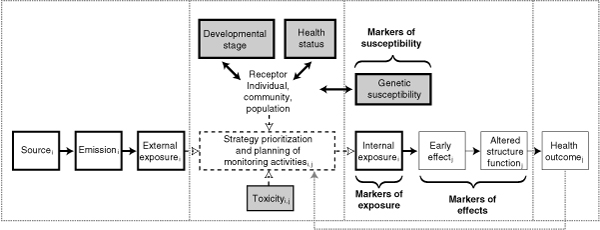
Biomonitoring framework to support the complex interaction between environmental quality and human health quality.

Results from the human biomonitoring program will feedback into the prioritization of the monitoring strategy (cf. Figure 2), so that new knowledge concerning the links between environmental quality and health outcome are made available to secure future optimizations of the biomonitoring strategy.

### The science-policy interface of the program

The hypothesis-driven risk scenario analysis will be based on integrated use of e.g. emission data, product information data, epidemiological data, air and drinking water quality data combined with available knowledge on possible human health effects. As such the risk scenario analysis is aims to cover all aspects of the DPSEEA (Driving forces – Pressures – State – Exposure – Effects – Actions) chain [12].

DPSEEA defines driving forces (D), that lead to pressures on the environment (P), which in turn change the state of the environment (S), resulting in human exposures (E) and in turn health effects (E). Actions (A) may then be taken at any point in this chain to mitigate or avoid unwanted health effects [12]. The structure illustrated above supports the development of information systems as well as the compilation of adequate political measures.

Environmental factors that induce health effects need to be investigated in order to support the selection of system actions with respect to identified direct and indirect environmental exposure sources. Identification of source-emission-exposure relationships is crucial for the response system within the DPSEEA framework to be operational for preventive actions for future protection of integrated environmental and human health aspects.

### Dataflow within integrated environment and health monitoring

For bridging the gaps in science-based knowledge of the health effects caused by environmental stressors, biomonitoring activities need to include environmental exposures, genetic susceptibility, diseases and/or disorders. To support further targeted policy actions for preventing and/or reducing diseases related to environmental stressors, human biomonitoring activities need to be designed in response to hypothesis testing of existing relations. This should also include available and emerging technologies for increasing the understanding of mechanisms causing unwanted health effects and routes of exposure related to specific environmental factors, i.e. the E-E part of the DPSEEA.

Such hypothesis testing will take place by prioritization of the human biomonitoring strategy and design of monitoring activities according to existing knowledge on sources, emissions, external exposures, toxicity of chemical exposures, susceptibilities of population groups to different types of diseases. Data flow and integration of new and existing knowledge as part of human biomonitoring framework is illustrated in Figure 2, which is described in the following paragraphs.

The boxes with black bold borders illustrate elements of an aggregated and cumulated exposure scenario (the source-emission-exposure link) and internal exposure, or biomarkers of exposure, as part of the human biomonitoring activities. The description of exposure scenarios includes identification of source and use categories, emissions and exposures. Additional chemical-specific properties may be taken into account addressing critical exposure routes, as has for example been carried out for BRFs in which case unintentional ingestion of dust is suggested at least of similar importance to food intake [25]. Likewise, existing fate models may be part of the exposure description of a given scenario type. External exposure monitoring is included in the biomonitoring activities where these data are needed to quantify the internal/external body exposure ratio as the combined monitoring high risk exposure route concentrations, and biomonitoring is essential for realistic human health impact assessment.

The grey shaded boxes with black bold borders represent cohort characteristics central for the hypothesis to be tested; a central element is genetic susceptibility biomarkers as part of the human biomonitoring activities.

The boxes with regular borders represent biomarkers of effects to be included in the human biomonitoring activities and human health outcome, which is part of the human health impact assessment in Figure 1. Biomarkers of effects represent early warning indicators 'signalling events in biological systems or samples', representing both the environmental agent and the adverse health effect. Included are markers of 1) susceptibility, 2) past and present exposure, 3) adverse effects and 4) specific diseases.

The grey shaded box with regular border represents available chemical toxicity data and may include all kinds of toxicity data that may be used for an initial risk scenario description and i.e. planning of monitoring activities. The box includes the use of classification and grouping of chemicals into similar modes of action.

To increase the transparency of how to support targeted policy for health protection, prevention or remedial actions, the links between scientific diagnostic health effects science and regulatory toxicity need to be addressed. Refined hypotheses are developed based on a national environmental and human health information system, giving an overview of research related to human health diseases suspected to be caused by chemical stressors.

The hypothesis is tested based on external and internal measurements of chemical exposure, biomarkers of exposure, susceptibility and effects. The hypothesis can be refined by hypothesis testing biomarkers of effects to the prioritized health effects (specific or non-specific adverse health effects) as illustrated by the iterative arrow from 'Human Health Impact Assessment' to 'Hypothesis driven risk scenario Analysis' in Figure 1 – analogue, the arrow from 'health outcome' to the 'strategy prioritization and planning of monitoring activities' in Figure 2.

## Discussion

Basic requirements for establishing a human biomonitoring program at the national and European level, described in this paper, are based on the recommendations of various international agencies. The important parameters for an optimal biomonitoring program include: selection of chemicals, exposure scenario and biological indicators (early warning indicators), which depend upon the target endpoint under investigation. It is important to develop a methodologically well-structured framework for biomonitoring activities that are able to support future optimal hypothesis testing of relationships between chemical exposure profiles and health effects. The target population groups and subgroups, chemical pollution in a geographical area and the genetic susceptibility of the population under study may affect selection of toxicological endpoints, and thereby the selection of chemicals and biomarkers may also be affected. Thus, the prioritization of chemicals and biomarkers both at national and European level will be required for optimal human biomonitoring in Europe. The experiences from a pilot project on European HBM would be able to establish scientifically-based selection criteria for various parameters. BFRs have been suggested as an important and optimal chemical group to include in the extended scenario for monitoring purposes and, based on existing data on external and internal exposure, the identification of near-field source contributions to exposures in aggregate. Such a pilot project would also indicate other important factors which should be under strict control when an HBM is being established in different Member States. This would contribute to harmonized HBM in Europe.

## Competing interests

The authors declare that they have no competing interests.

## Authors' contributions

The authors of the paper are all members of a Danish working group that has made an effort to gather all national main actors within human biomonitoring research in Denmark. The group prepared an expression of interest for a Danish human biomonitoring programme to the relevant authorities and suggested a Danish human biomonitoring programme at the same scale as the existing National Monitoring and Assessment Programme for the Aquatic and Terrestrial Environment (NOVANA).

Marianne Thomsen has taken the initiative to write this paper based on the experiences from the working group and scientific projects such as e.g. NoMiracle, state of environment and health reporting projects and her involvement in the development of a coupled monitoring and assessment strategy for NOVANA. Hanne Bach, Peter B. Sørensen and Patrik Fauser have been involved in the same projects and contributed mainly in the early phase of this paper, with input on the structure of the paper and, more specifically, a cause-effect framework.

Lisbeth E. Knudsen contributed to the manuscript with her insights into human biomonitoring activities on the national and European level, among others from participation in the EU network "Expert team to support biomonitoring in Europe (ESBIO)". Lisbeth E. Knudsen, Katrin Vorkamp, Marie Frederiksen and Marianne Thomsen have been involved in related human biomonitoring activities with focus on brominated flame retardants. Katrin Vorkamp has also contributed with experience from NOVANA and the Arctic Monitoring and Assessment Programme (AMAP).

Suresh Rastogi has long term experience with near-field exposure from consumer products. Eva Bonefeld-Jørgensen has likewise been active within human biomonitoring for many years, among others within the Human Health Programme under AMAP. Teddy Krongaard coordinated the Danish expression of interest. All authors have contributed to the manuscript with discussion and comments.
